# Artificial Intelligence and Its Role in the Diagnosis and Prediction of Adverse Events in Acute Coronary Syndrome: A Narrative Review of the Literature

**DOI:** 10.3390/life15040515

**Published:** 2025-03-21

**Authors:** Andrea Mariani, Carmen Anna Maria Spaccarotella, Francesco Saverio Rea, Anna Franzone, Raffaele Piccolo, Domenico Simone Castiello, Ciro Indolfi, Giovanni Esposito

**Affiliations:** 1 Department of Advanced Biomedical Sciences, University of Naples “Federico II”, Via Sergio Pansini 5, 80131 Naples, Italy; dr.marianiandrea@gmail.com (A.M.); carmenannamaria.spaccarotella@unina.it (C.A.M.S.); francescorea77@gmail.com (F.S.R.); anna.franzone@unina.it (A.F.); raffaele.piccolo@unina.it (R.P.); ds.castiello@gmail.com (D.S.C.); espogiov@unina.it (G.E.); 2 Department of Pharmacy, Health and Nutritional Sciences, University of Calabria, Via Pietro Bucci, Arcavacata, 87036 Rende, CS, Italy

**Keywords:** artificial intelligence, acute coronary syndrome, narrative review, machine learning

## Abstract

Acute coronary syndrome (ACS) is a global health concern that requires rapid and accurate diagnosis for timely intervention and better patient outcomes. With the emergence of Artificial Intelligence (AI), significant advancements have been made in improving diagnostic accuracy, efficiency, and risk stratification in ACS management. This narrative review examines the current landscape of AI applications in ACS diagnosis and risk stratification, emphasizing key methodologies, technical and clinical implementation challenges, and also possible future research directions. Moreover, unlike previous reviews, this paper also focuses on ethical and legal issues and the feasibility of clinical applications.

## 1. Introduction

Cardiovascular disease (CVD) remains the most common cause of death worldwide. Using the latest data available, CVD caused more than 3.8 million deaths per year across Europe, accounting for just under 1.76 million deaths in men and more than 2 million deaths in women. Between these almost 4 million deaths, ischemic heart disease (IHD) was the most common cause, accounting for 47% of all CVD deaths in men and 40% of all CVD deaths in women [1]. In 2019, there were an estimated 5.8 million new cases of IHD in the 57 ESC member countries and an estimated 47.6 million people living with IHD in the same countries. The European Heart Network has estimated that CVDs cost the European Union (EU) economy more than EUR 200 billion a year, of which more than half is due to healthcare costs [2]. In this scenario, the burden of mortality, morbidity, and healthcare costs of IHD could be mitigated by the early and timely diagnosis of its most dangerous and feared manifestation, acute coronary syndrome (ACS), which includes myocardial infarction with or without ST-segment elevation (STEMI and NSTEMI) and unstable angina [3,4]. However, the diagnostic process for ACS is challenging, as only 5.1% of patients presenting with chest pain are diagnosed with an ACS and several cardiac or non-cardiac conditions should be considered in the differential diagnosis of acute chest pain as part of the clinical assessment [5]. Furthermore, troponin elevation is not exclusive to ACS; it can occur in many other conditions, such as heart failure, rhythm disorders, or even in non-cardiac conditions such as infections, autoimmune diseases, chronic kidney disease, or alcohol and drug abuse [6]. Because of the complexity of diagnosis, the limitation of clinical assessment, and the potential socio-economic impacts of improving diagnosis, there is ongoing research for techniques and strategies that could improve diagnosis and treatment of ACS. Among these, artificial intelligence (AI) has an emerging role. AI is a broad term for complex computerized analytical programs and algorithms capable of using human-like intelligence and performing tasks commonly associated with intelligent beings [7]. This review explores the current literature on the use of AI in diagnosing ACS and the prediction of MACE (Major Adverse Cardiovascular Events) and all-cause mortality in ACS.

## 2. Materials and Methods

This narrative review was written following SANRA (Scale for the Assessment of Narrative Review Articles) guidelines [8]. A comprehensive search of electronic databases, including PubMed, Web of Science (WOS), Excerpta Medica database (EMBASE), and Google Scholar, was conducted to identify relevant studies published between 2014 and 2024. We also investigated studies listed as references in the selected articles. The following searched terms or phrases were used: “artificial intelligence and cardiology”; “artificial intelligence and acute coronary syndrome”; “artificial intelligence and coronary artery disease”; “artificial intelligence and myocardial infarction”; “artificial intelligence and STEMI diagnosis”; “artificial intelligence and NSTEMI diagnosis” “artificial intelligence and interventional cardiology”; “machine learning and cardiology”; “machine learning and acute coronary syndrome”; “machine learning and coronary artery disease”; “machine learning and myocardial infarction”; “machine learning and STEMI diagnosis”; “machine learning and NSTEMI diagnosis”; “machine learning and interventional cardiology”; “artificial intelligence and MACE prediction in ACS”; and “machine learning and mortality prediction in ACS”.

**Figure 1:**
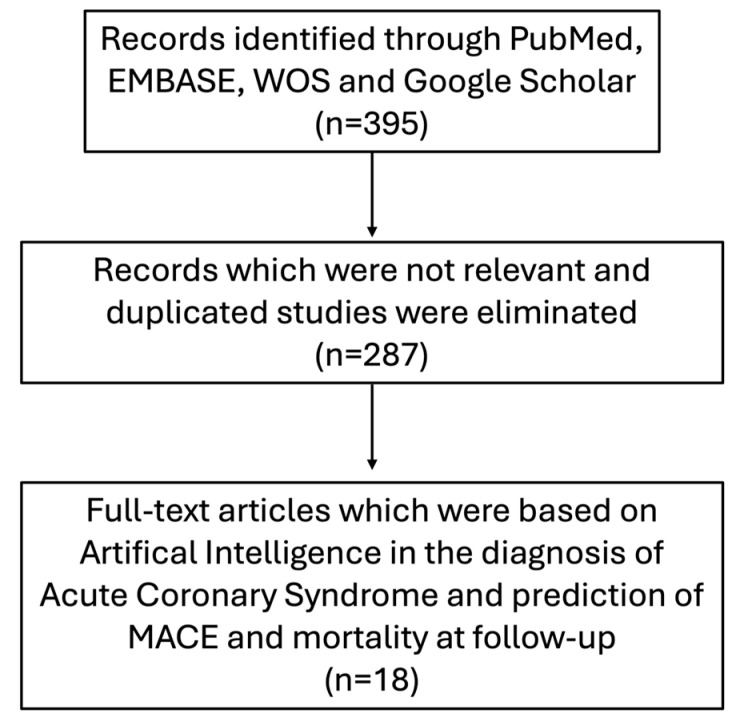
Flow diagram of the search process.

Figure 1 provides a flow diagram of the search process. Table 1 and Table 2 summarize the principal features of the studies evaluated in this review.

## 3. Diagnosis of ACS and Pitfalls

ACS encompasses a group of conditions in which there is an abrupt limitation of blood flow to the heart, including patients presenting with recent changes in clinical symptoms (e.g., angina), with or without changes in the 12-lead electrocardiogram (ECG) and with or without acute increases in cardiac troponin (cTn) concentrations. ACS comprises the following three distinct entities: ST-elevation myocardial infarction (STEMI), non-ST elevation myocardial infarction (NSTEMI), and unstable angina (UA) [9]. According to the fourth universal definition, myocardial infarction is diagnosed when there is clinical evidence of myocardial ischemia (symptoms and/or ECG changes) associated with acute myocardial injury defined as a rise and/or fall of cTn values with at least one above the 99th percentile upper reference limit; on the other hand, unstable angina is defined as myocardial ischemia at rest or after mild exertion in the absence of myocardial injury [10]. ACS is often symptomatic in its most typical presentation with retrosternal chest pain/chest discomfort. However, about 40% of men and 48% of women have non-specific symptoms such as dyspnea or diaphoresis (these are defined as “anginal equivalents”) [11]. According to 2023 European Society of Cardiology (ESC) guidelines, the current protocol to detect ACS in a patient with clinical presentation suggestive of ongoing myocardial ischemia uses electrocardiograms and serum troponin. ECG is the first-line tool in the assessment of the patient with suspected ACS and is recommended to be performed within 10 min of the first medical contact [9,12]. ECG can categorize patients with suspected ACS into two working diagnoses: STEMI—in which there is persistent ST-segment elevation at the J-point in at least two contiguous leads—and NSTE-ACS—in which the ECG may be normal or other abnormalities may be present, such as transient ST elevation, ST depression, and T wave changes [9]. For patients with an operative diagnosis of STEMI, an immediate (within 120 min of the ECG-based diagnosis) reperfusion strategy with primary percutaneous coronary intervention (PPCI) is recommended; this strategy can reduce mortality up to 9%, and each 30 min delay increases one-year mortality by 7.5% [9,11,13]. Therefore, especially in STEMI patients, correct and timely diagnosis is critical.

In patients where an immediate invasive strategy is not required, like those with suspected NSTEMI without very-high-risk features (e.g., hemodynamic instability, cardiogenic shock, and mechanical complications) or unstable angina, biomarkers play a complementary role, and the use of high-sensitivity cardiac troponin (hs-cTn) is highly recommended [9,14]. The ESC recommends the use of a rule-in/rule-out algorithm based on a baseline measurement (0 h) and a second measurement at 1 or 2 h from the first one (0 h/1 h and 0 h/2 h), which can safely detect or exclude MI in a shortened time compared to previous algorithms, due to the high sensitivity and diagnostic accuracy of hs-cTn [9,15].

Even though the diagnostic pathway of ACS is well codified, it remains full of pitfalls. First, even for experienced physicians, ECG interpretation in the setting of ACS can be challenging. Previous studies showed a pooled accuracy of 68.5% in ECG interpretation for practicing physicians, with the rate increasing to 74.9% for cardiologists [16,17]. In addition, some ECG patterns, such as left bundle branch block (LBBB), right bundle branch block (RBBB), and paced rhythm, can preclude an accurate assessment of ST-segment elevation, making a correct ECG interpretation difficult. In patients with LBBB, Sgarbossa criteria and other scores may improve diagnostic accuracy. Nonetheless, only one-third of patients who attend the emergency department with signs and symptoms suggestive of MI and LBBB will turn out to have an acute myocardial infarction (AMI) as the final diagnosis. Hence, as reported in the latest 2023 ESC guidelines, patients with these ECG patterns and clinical features highly suspicious of ongoing myocardial ischemia should be managed similarly to those with clear ST elevation and undergo immediate invasive coronary angiography, regardless of whether the bundle branch block is previously known [9,18,19].

Although complete vessel occlusion usually presents with ST-segment elevation, more than 25% of patients presenting with NSTE-ACS may reveal complete culprit vessel occlusion on subsequent angiography, with a predilection for inferolateral localization; such patients may represent a higher risk group between NSTEMI patients with higher risks of mortality and MACE; hence, they need to be rapidly recognized and referred promptly for immediate reperfusion strategy, in the same way as STEMI patients [20,21]. However, due to the absence of a clear ST-segment elevation, it is not always easy to rapidly recognize this subgroup of patients. Indeed, in the literature, some ECG patterns associated with total vessel occlusion in the absence of ST-segment elevation should be part of every cardiologist and emergency physician’s ECG knowledge to avoid late referral to the catheterization laboratory and misdiagnosis with subsequent worse outcomes [9,22,23].

It is not only ECG interpretation that can complicate the ACS diagnostic process, but also the analysis and evaluation of cardiac troponins. Troponins are not just released into circulation during myocardial ischemia, but are an index of myocardial damage from any cause (myocardial injury) and can be elevated for multiple cardiac and non-cardiac conditions other than MI. Moreover, four clinical variables are described as significantly affecting hs-cTn concentrations beyond the presence or absence of MI: age, renal dysfunction, time from chest pain onset, and, to a lesser extent, sex [24,25,26]. Nevertheless, the introduction of high sensitivity cardiac troponin has increased cardiac troponin diagnostic sensitivity and accuracy, allowing a more rapid MI rule-in and rule-out, and shortening the “troponin-blind” interval, leading to earlier detection of MI; unfortunately, this improved diagnostic sensitivity has been accompanied by a decline in specificity for the diagnosis of type 1 MI, identifying type 2 MI more, along with other acute or chronic conditions associated with an increase in circulating cTn, leading to a rise in NSTEMI diagnoses. Therefore, the use of hs-cTn allows early detection not exclusively of MI, but also of other cardiac and non-cardiac conditions; differential diagnosis between them and prompt identification of type 1 MI with subsequent immediate reperfusion therapy represent still a challenge for physicians [14,27,28].

## 4. AI and Its Role in the Diagnosis of ACS

AI includes any technique that allows computer systems to mimic human intelligence and behavior. Machine Learning is a subset of AI that uses statistical methods and specific algorithms to enable machines to learn from data and improve with experience. Once the ML algorithm is trained with data, the ML model can be provided with input from large datasets. The output will be a predictive model based on the data that trained the model and on error feedback. One of the most used methodologies is deep learning (DL), a subfield of ML, inspired by the complexity of human brain and based on artificial neural networks (ANNs) in which multiple layers of processing are used to extract progressively higher-level features from data, showing an advanced capability to process large amounts of complex information [29]. In recent years, AI has been emerging as a rapidly expanding field in medicine, particularly in cardiology, where it has been channeled toward enhancing diagnostic and prognostic capabilities, utilizing various resources such as hospital datasets, electrocardiograms, and echocardiograms. Diagnosis and prediction are the most common functions of AI models in the cardiovascular field. In AI, intense learning has demonstrated robust applications across cardiovascular subspecialties, like ischemic heart disease. It could improve the diagnostic pathway of ACS by processing and integrating medical history, ECG, cardiac markers, and cardiac imaging data (Table 1) [30].

**Table 1:** Studies analyzing artificial intelligence in the diagnosis of acute coronary syndrome.

First Author, Year of Publication, Reference No.	No. of Patients	Machine Learning Models	Data	Application	Accuracy/ AUROC/ F_1_-Score *
Al Zaiti et al., 2020 [31]	n = 1244	LR GBM ANN	12-lead ECG	Diagnosis of ACS Diagnosis of NSTE-ACS	0.76/0.82/- 0.74/0.78/- (ML-fusion)
Chen et al., 2022 [32]	n = 275	CNN-LSTM	12-lead ECG	Diagnosis of STEMI	0.99/0.99/0.91
Zhao et al., 2020 [33]	n = 8238	Res-Net	12-lead ECG	Diagnosis of STEMI	0.94/0.97/0.93
Choi et al., 2022 [34]	n = 187	CNN	12-lead ECG	Diagnosis of STEMI	0.86/0.91/-
Herman et al., 2024, [35]	n = 12,765	DNN	12-lead ECG	Diagnosis of OMI	0.91/0.94/-
Liu et al., 2021 [36]	n = 77,799	DLM	12-lead ECG 12-lead ECG + hs-cTnI	Diagnosis of STEMI Diagnosis of NSTEMI	0.96/0.97/- 0.95/0.98/-
Wu et al., 2019 [37]	n = 268	ANN	Clinical, laboratory and 12-lead ECG data	Diagnosis of NSTEMI	0.93/0.98/-
Qin et al., 2023 [38]	n = 2878	SVM XGBoost RF NB LR GBM	Clinical, laboratory and 12-lead ECG data	Diagnosis of NSTEMI	0.95/0.97/0.96 (XGBoost)
Berikol et al., 2016 [39]	n = 228	SVM NB LR	Clinical, laboratory, echocardiogra-phic and 12-lead ECG data	Diagnosis of ACS	0.99/-/1 (SVM)
Than et al., 2019 [40]	n = 11,011	GBM	Clinical data and hs-cTnI	Diagnosis of type 1 MI	0.97/0.96/-
Doudedis et al., 2023 [41]	n = 20,324	XGBoost	Clinical, laboratory data and hs-cTnI	Diagnosis of MI	0.94/0.95/-
Kayvanpour et al., 2021 [42]	n = 148	ANN	Micro-RNAs (10 miRNA analyzed)	Diagnosis of ACS	0.96/0.99/-

* Every accuracy, AUROC, and F_1_-score value has been rounded to two decimal places. A single dash instead of a value indicates a missing value. In cases where multiple ML algorithms were evaluated, we choose to report only parameters of the best performing one (e.g., [38]). Abbreviations (in order of appearance): AUROC—area under the receiver operating curve; LR—logistic regression; GBM—gradient boosting machine; ANN—Artificial Neural Network; ACS—acute coronary syndrome; STEMI—ST-elevation myocardial infarction; NSTEMI—Non-ST-elevation myocardial infarction; CNN-LSTM—convolutional neural network and long short-term memory; Res-Net—Residual Network; CNN—convolutional neural network; DNN—Deep Neural Network; OMI—Occlusion Myocardial Infarction; DLM—Deep Learning Model; hs-cTnI—high sensitivity cardiac Troponin I; SVM—support vector machine; XGBoost—eXtreme gradient boosting; RF—random forest; NB—Naïve Bayesian.

Several authors have proposed using ML-based methods to analyze ECG features to achieve accurate and timely diagnosis of ACS. Al Zaiti et al. trained an ANN on 745 patients, and externally validated it on 499 patients, to identify, from a pre-hospital 12-lead ECG, ongoing myocardial ischemia between patients who had called the local emergency number (911) for acute chest pain. From the analysis of more than one thousand ECGs, the ANN model outperformed commercial ECG algorithms and expert clinicians in detecting STEMI and NSTE-ACS events, with a good AUROC (area under the receiver operating characteristic curve) of 0.82, a high NPV (negative predictive value) of 94% and a reasonable specificity of 76%, resulting in a 37% gain in sensitivity compared to experienced clinicians and 52% gain in sensitivity compared to commercial ECG algorithms, corresponding to a net reclassification improvement (NRI) of 0.19 (95% CI 0.06–0.31) and 0.30 (95% CI 0.19–0.41), respectively [31]. Chen et al. proposed an AI model (trained and tested on 6914 ECGs) combining a convolutional neural network and long short-term memory (CNN-LSTM) to diagnose STEMI using 12-lead mini-ECG devices equipped in ambulance vehicles in central Taiwan; ECG signals were transmitted to the AI center at China Medical University Hospital and classified as STEMI or non-STEMI. The AI’s response time was 37.2 ± 11.3 s, which was shorter than the online physicians’ response time (113.2 ± 369.4 s, *p* < 0.001); during the study period, among 362 pre-hospital ECGs, the AI model promptly identified, with a calculated AUROC and accuracy of 99%, 10 STEMI patients who underwent PPCI with a median contact-to-door time of 18.5 min, demonstrating also that this approach can help minimize avoidable delays for STEMI patients [32]. Further studies compared the efficiency of AI algorithms versus physicians in diagnosing STEMI from solely electrocardiographic data. Zhao et al. showed that an AI algorithm for STEMI detection (trained on 36,466,080 ECGs and externally validated on 2441 ECGs) made the right diagnosis with an AUROC of 0.997 and high accuracy of 94% and precision (F1 score 0.9375, a machine learning evaluation metric that measures a model’s accuracy), which resulted in a much higher accuracy than that of a group of 15 cardiologists in a comparative test of 100 ECGs (accuracy of 80.3% and F1 score of 0.7865) [33]. Similar results were achieved by Choi et al., comparing an AI algorithm (trained on 96,925 ECGs) for the detection of STEMI through an app on researchers’ smartphones (QCG—Quantitative ECG) with diagnosis made by physicians (cardiologists and emergency physicians); however, in this study, the CNN algorithm has been tested not only on the original waveform images, but also on printed ECGs and ECG images on PC screen. Once more, AI outperformed physicians on 187 ECGs with higher AUC (0.919 vs. 0.856, *p* = 0.004) and higher sensitivity, specificity, PPV (positive predictive value), and NPV, indicating that it could be a practical and prompt system for STEMI detection using only a smartphone [34].

As mentioned earlier, one-third of patients with NSTEMI have an acutely occluded culprit coronary artery, presenting with occlusion myocardial infarction (OMI) and, thus, with a worse prognosis than the other NSTEMI patients. Herman et al. developed an AI model on 18,616 ECGs that detects OMI on standard 12-lead ECGs of patients with suspected ACS in less time and with superior accuracy (AUROC 0.938) compared to cardiologists applying ECG STEMI criteria (validated on 3254 ECGs), whilst it resulted in comparable accuracy and timing relative to a cardiologist who was an expert in ECG reading. Therefore, this DLM (deep-learning model) could streamline the timely referral of ACS patients at risk of poor outcomes, like patients with NSTEMI and occluded culprit coronary artery that should be revascularized within 2 h as STEMI patients [35].

To further increase the diagnostic capability of AI algorithms, some authors have integrated ECG data with other parameters that correlate with ACS. For instance, Liu et al. developed (trained on 58,056 patients, validated on 19,743 patients) a DLM that demonstrated high, and superior to physicians, diagnostic ability for STEMI detection from only ECG (AUC = 0.997; sensitivity = 98.4%; specificity = 96.9%), whereas it emerged that, for the detection of NSTEMI, the AUC was higher (0.978) than that of cTnI (0.950) or the DLM (0.877) alone, when it has been considered the combination of DLM with conventional cardiac troponin I [36]. Another study has shown that an ANN model (trained on 159 patients, internally validated on 53 patients, and tested on 56 patients) has high AUROC (0.984), sensitivity (90.91%), specificity (93.33%) and accuracy (92.86%) in identifying NSTEMI from a set of clinical, laboratory, and electrocardiographic data (without the analysis of ST segment). Thus, it could emerge as a potential tool for the diagnosis of ongoing ischemia in patients at risk of ACS with no clear ST-segment elevation [37]. In addition, Qin et al. compared six different ML models, encompassing 2878 patients, in assisting the diagnosis of NSTEMI on the basis of clinical, laboratory, and electrocardiographic data (ST-segment in lead aVR); it emerged that the extreme gradient boosting (XGboost) model performed best in terms of accuracy rate (95%) and precision (94%), highlighting its usefulness as an auxiliary tool to improve the accuracy of NSTEMI diagnosis (trained on 391 patients, internally validated on 85 patients, and tested on 225 patients) [38]. Moreover, Berikol et al. proposed a support vector machine (SVM) system for machine learning, trained and tested on 228 patients, to help clinicians diagnosing solely acute coronary syndrome, not specifically STEMI or NSTEMI, through the analysis of clinical, laboratory, electrocardiographic, and echocardiographic data; it proved to have a very high accuracy of almost 100% (99.13%) and high sensitivity and specificity (98.22% and 100%, respectively) [39].

Furthermore, it has been demonstrated that diagnosis of MI could be safely made by AI algorithms even without ECG, but only by evaluating other elements like laboratory and clinical data. In an interesting paper published in *Circulation*, a machine learning algorithm (gradient boosting machine—GBM) called myocardial-ischemic-injury-index (MI^3^), that incorporates age, sex, and paired hs-cTnI concentrations (at least two measurements), was trained on 3013 patients and tested on 7998 patients for the diagnosis of type 1 myocardial infarction; MI^3^ performed better in predicting the likelihood of MI than the ESC 0/3 *h* pathway and the 99th percentile of hs-cTnI at any time point, with a very high AUROC of 0.963, leading to a faster and more reliable diagnosis [40]. In a similar paper published more recently in *Nature*, Doudesis et al. developed, on a training set of 10,038 patients, an ML model (XGboost) named CoDE-ACS (Collaboration for the Diagnosis and Evaluation of Acute Coronary Syndrome), that integrates cardiac troponin concentrations at presentation with relevant clinical features that either influence troponin levels (age, sex, renal function, time from symptoms onset, the presence of chest pain, and hemoglobin) or increase pre-test probability (known IHD, hyperlipidemia, heart rate, systolic blood pressure, Killip class, and ECG signs of myocardial ischemia); this clinical decision support system outperformed, on the validation set of 10,286 patients, guideline-recommended protocols like the ESC 0/1 h, revealing higher AUROC (0.953), PPV and NPV, even in the subgroups where all the other protocols produced more false positives and negatives (e.g., women, elderly, those with renal impairment or those who presented early to medical attention following the onset of symptoms). Furthermore, the score (0–100) computed by CoDE-ACS representing the patient’s probability of MI has been demonstrated to correlate with significant outcomes as cardiac death and all-cause mortality at 30 days and 1 year (log-rank test *p* < 0.001). Hence, if adopted in practice, CoDE-ACS could reduce time spent in emergency departments, prevent unnecessary hospital admissions, and improve the early treatment of myocardial infarction, with benefits for both patients and healthcare providers [41].

Finally, another possible and innovative application of ML models has been tested by a group of authors that developed, in 121 blood samples (training sets), an ANN model incorporating 34 validated micro-RNAs (miRNAs) for the diagnosis of ACS, achieving excellent classification results on 13 testing samples; through the analysis of 10 selected miRNAs from a single blood sample, this ANN model turned out to be superior to the one-point hsTnT value, with an AUROC of 99%, an accuracy of 96%, a sensitivity of 95%, and a specificity of 96%, in the diagnosis of ACS [42].

## 5. AI and Its Role in Predicting Major Adverse Cardiac Events (MACE) in Patients with ACS

ACS poses a significant risk of adverse events to the patient even after successful treatment. Indeed, from a recent Swedish population-based cohort study, it emerged that by 1 year, 10.3% of ACS patients had experienced one of the analyzed MACE (CV death, MI, and ischemic stroke), with a significant increase in their incidence up to 28.6% at the end of the follow-up (4.7 years) [43]. Based on patients’ clinical features at admission, the Global Registry of Acute Coronary Events (GRACE) risk score is currently the most well-established and widely recommended risk stratification tool for patients presenting with ACS across international guidelines [9].

AI is emerging to aid diagnosis and risk stratification (Table 2). 

**Table 2:** Studies analyzing artificial intelligence predicting of MACE and all-cause mortality in acute coronary syndrome.

First Author, Year of Publication, Reference No.	No. of Patients	ML Models	Outcome Predicted	Performance (AUROC/F_1_-Score) *
Khera et al., 2021 [44]	n = 755,402	XGBoost ANN Meta-classifier	In-hospital mortality	XGBoost: 0.90/0.43 ANN: 0.88/0.41 Meta-classifier: 0.89/0.43
Hadanny et al., 2021 [45]	n = 25,709	RF	30-day mortality	RF: 0.80/-
Sherazi et al., 2020 [46]	n = 8227	GBM DNN RF GLM	1-year mortality	GBM: 0.90/0.97 DNN: 0.90/0.95 RF: 0.89/0.96 GLM: 0.87/0.96
Sherazi et al., 2020 [47]	n = 11,189	SVE ET RF GBM	MACE	SVE: 0.99/0.91 ET: 0.99/0.90 RF: 0.98/0.90 GBM: 0.98/0.85
D’Ascenzo et al., 2021 [48]	n = 19,826	PRAISE score (AdaBoost)	1-year mortality Recurrent MI Major bleeding	AdaBoost: 0.92/- AdaBoost: 0.81/- AdaBoost: 0.86/-
Mohammad et al., 2022 [49]	n = 139,288	ANN	1-year mortality 1-year HF hospitalization	ANN: 0.84/- ANN: 0.78/-
Lee et al., 2021 [50]	n = 14,183	RF SVM XGBoost Lasso LR Ridge LR Elastic net LR	In-hospital mortality 3-month mortality 1-year mortality	**STEMI—In-hospital mortality** RF 0.92/0.09 SVM 0.87/0.07 XGBoost 0.94/0.11 Lasso LR 0.92/0.12 Ridge LR 0.92/0.08 Elastic net LR 0.92/0.12
				**STEMI—1-year mortality** RF 0.77/0.03 SVM 0.69/0.02 XGBoost 0.80/0.04 Lasso LR 0.79/0.05 Ridge LR 0.79/0.04 Elastic net LR 0.79/0.04
				**NSTEMI—In-hospital mortality** RF 0.92/0.10 SVM 0.85/0.06 XGBoost 0.91/0.10 Lasso LR 0.92/0.01 Ridge LR 0.92/0.10 Elastic net LR 0.92/0.10
				**NSTEMI—1-year mortality** RF 0.79/0.10 SVM 0.72/0.08 XGBoost 0.81/0.11 Lasso RF 0.82/0.10 Ridge RF 0.81/0.10 Elastic net RF 0.81/0.10

* When more than one system is tested, the best performing ML algorithm in each study is underlined. All the AUROC and F_1_-scores reported are the ones obtained in the external validation cohort, and not in the testing or training dataset. Every AUROC and F1-score value has been rounded to two decimal places. A single dash instead of a value indicates a missing value. Abbreviations (in order of appearance): AUROC—area under the receiver operating curve; XGBoost—eXtreme gradient boosting; ANN—artificial neural network; GBM—gradient boosting machine; DNN—Deep Neural Network; RF—random forest; GLM—Generalized Linear Model; STEMI—ST-elevation myocardial infarction; NSTEMI—Non-ST-elevation myocardial infarction; SVE—Soft Voting Ensemble; ET—extra tree; MACE—Major Adverse Cardiovascular Events; SVM—support vector machine; AdaBoost—Adaptive Boosting; LR—logistic regression.

Various ML models have been trained on 564,918 patients and tested on 190,484 patients to predict the in-hospital mortality (short-term prediction) of patients with MI. This is the only setting explored where AI does not outperform regular statistical models; two of the tested models, XGboost and meta-classifier, improved only calibration across the risk spectrum, reclassifying one in every four patients, deemed moderate or high risk for death with logistic regression, as low risk, whereas no difference from logistic regression on predictions of in-hospital mortality was noted [44]. Hadanny et al., in a retrospective, supervised learning, data mining study, developed, on the Acute Coronary Syndrome Israeli Survey (ACSIS) registry (training set: 2782 patients) and on the Myocardial Ischemia National Audit Project (MINAP) (validation set: 22,963 patients), a random forest (RF) predictive model for 30-day mortality (short-term prediction) after STEMI and compared it with the commonly used GRACE score; through this study, the authors demonstrated that such an ML-model can outperform known risk scores (AUC of RF 0.804 vs. AUC of GRACE score 0.764, *p* = 0.008) [45]. In addition, Sherazi et al. used a dataset of 8227 patients (6606 for training, 1621 for testing) from the Korea Acute Myocardial Infarction Registry (KAMIR) to develop and compare four different machine learning models in prediction of 1-year mortality (long-term prediction) after hospital discharge in patients admitted for ACS; from this comparison, the GBM model (AUC 0.898, accuracy 94%) emerged as superior to the other ones, and each ML-algorithm was better when compared with the GRACE score (AUC 0.810, accuracy 90%) [46]. In a similar work from the same group of authors, four ML models (GBM, RF, soft-voting ensemble classifier, and extra tree) were compared (7832 patients for training, 3357 patients for testing) for early prediction of 2-year MACE (cardiac death, non-cardiac death, MI, PCI, and coronary artery bypass grafting) in patients suffering with ACS (long-term prediction); the soft-voting ensemble classifier (SVE) was found to be significantly superior to the other machine learning models, with a calculated AUC of 0.99 [47]. D’Ascenzo et al. developed a machine learning-based model (Adaptive Boosting) using 19,826 patients (training set), known as the PRAISE score, which showed on a cohort of 3444 patients accurate discriminatory capacity in predicting 1-year all-cause mortality (long-term prediction), recurrent myocardial infarction, and major bleeding (defined as Bleeding Academic Research Consortium type 3 or 5) in patients discharged after ACS hospitalization [48]. Moreover, in a population-based study, data from the nationwide SWEDEHEART registry was used to train (11,1558 patients) and test (27,730 patients) an artificial neural network algorithm to predict, in 30,971 patients with ACS from the Western Denmark Heart Registry, 1-year all-cause mortality and 1-year heart failure hospitalizations (long-term predictions). This model was better than the GRACE score in predicting 1-year mortality (AUROC of extended model 0.87 vs. 0.78) and was found to be a reliable system to estimate HF hospitalization within 1 year (AUROC of extended model 0.84 vs. 0.67) [49].

Finally, in an interesting study published in Nature’s *Scientific Reports*, various ML-models (XGboost, RF, Lasso regression, elastic net regression, Ridge regression, and SVM) have been investigated in estimating in-hospital (short-term prediction), 3-month and 12-month mortality (long-term predictions) within a cohort of 14,183 patients with AMI (11,346 training and 2837 testing), also comparing their performance in the two subgroups of myocardial infarction with or without ST-segment elevation. It turned out that the performance of ML models in predicting the mortality of patients, at any time, with STEMI was comparable to that of the traditional models (GRACE, TIMI, ACTION-GWTG, and their modified versions). In contrast, the AUCs of the ML models for NSTEMI were superior to those of traditional models in predicting long-term mortality. This study suggests that the ML algorithms could overcome the performance of conventional predictive models in ACS especially in specific settings (NSTEMI patients and long-term mortality) [50]. Hence, AI could definitely help, through the prediction of short- and long-term outcomes of ACS, to better manage higher-risk patients in ICU (intensive care unit), by reserving longer lengths of stay them, along with more aggressive secondary preventive medications, and referrals to cardiac rehabilitation (where these patients benefit more than lower-risk patients), whereas early discharge and less aggressive therapies could be addressed to lower-risk patients [51].

## 6. Challenges and Future Directions

Despite the enormous potential of ML-based artificial intelligence systems in the diagnostic and prognostic pathway of ACS, several potential difficulties must be pointed out, and they could be categorized as follows:

1. Technical challenges: First, training neural networks requires a large amount of data to be accessible, and, in this regard, lacking data remains a challenge. Data collection and storage remain challenging, requiring innovative tools and collaborations among multiple centers to acquire enough data to train high-performance models. Furthermore, deep learning allows deep relationships between data to be formulated. Still, this form of learning is highly dependent on the quality and reliability of the data to which it is exposed: any pre-existing systematic errors in the source could lead to a risk of perpetuating them. Neural networks are highly vulnerable to minimal perturbations in the data (black box nature). For example, the change of a few pixels in an input image, imperceptible to the human observer, could lead a well-validated CNN to make a radical error in data classification, resulting in an incorrect output. The quality of data obtainable in clinical practice on which an ML model is tested and validated is a further issue: the models are often derived from high-quality databases with meticulously obtained ECGs, so the application of such obtained models in the emergency setting, where the quality of ECG acquisition is not always the best, could be a problem. Moreover, there is a concern that these algorithms may not be generalizable to diverse patient populations (e.g., different ethnicities) and thus will require more rigorous validation across healthcare systems.
2. Ethical and legal challenges: Data ownership remains another unresolved issue: the use of each patient’s data within the ML could be seen as a potential privacy violation; the exchange of patient data between various research centers around the world is a complex process that raises concerns about the security and protection of sensitive patient information that could be susceptible to cyber-attacks. It should also be considered that the use of AI in medical decision-making has not yet been legally defined and correctly regularized, leaving numerous debates open in multiple scenarios. For instance, in the event of misclassification by the AI model, in the absence of a precise regulation, the question of to whom the liability belongs must be addressed—to the physician using the model, to the programmer, or to AI itself—with the latter, nowadays, still not being recognized as a legal entity, thus necessitating more clarity by regulatory and health agencies (the AI Act from the European Union AI Office still pending).
3. Clinical implementation challenges: It is crucial to cautiously integrate these advanced tools with physician decision-making. While AI has shown promise in enhancing diagnostic accuracy, streamlining workflows, and offering prognostic forecasting, it cannot be seamlessly incorporated into our daily clinical practice. One of the primary hurdles is ensuring that AI tools are user-friendly and compatible with existing clinical infrastructure, such as Electronic Medical Records (EMRs) systems, to minimize disruptions and optimize efficiency. Moreover, AI’s possible role in decision-making raises concerns about the potential erosion of physicians’ clinical autonomy. To address these challenges, there is an urgent need for robust training programs that equip healthcare professionals with the skills to effectively use AI systems. These should include not only training on how to deal with AI tools, but also on how to critically assess and integrate AI recommendations into clinical practice. Physicians and other healthcare providers must understand the underlying algorithms, recognize their limitations, and develop the capability to make decisions that incorporate both human expertise and machine learning-based insights.

Future research directions should include an analysis of the cost-effectiveness and cost–benefit ratios of AI technology and its impact on daily clinical practice and, more importantly, on patient outcomes. They should validate AI models across diverse patient populations and healthcare environments to increase generalizability of each model. Furthermore, scientific societies and institutions, together with healthcare providers, should develop ethical guidelines for AI use in clinical practice and establish AI-assisted diagnostic protocols. Finally, novel data sources such as wearable devices and genetic information should also be explored to further refine ACS diagnosis and risk stratification algorithms.

## 7. Conclusions

AI is emerging as an up-and-coming tool in cardiovascular medicine and the diagnostic path of ACS. AI appears superior to physicians in interpreting and diagnosing ACS from ECG and clinical and laboratory data, demonstrating extraordinary ability to integrate multiple sources and heterogeneous information to reach a correct and definite diagnosis. In addition, AI has been shown to provide, in ACS patients, valuable support in predicting long-term mortality and MACE, often superior to traditional statistical models. With continued research and development, AI-driven approaches are poised to transform the landscape of ACS management, ultimately improving patient outcomes and reducing healthcare burdens. However, AI still has broad limitations regarding data bias, legal regulations, and integration with medical judgment that must be addressed before it can be commonly and universally applied in daily clinical practice. Hence, collaborative efforts among clinicians, researchers, industry stakeholders, and policymakers are essential to harness AI’s full potential in improving ACS care. Thus, until that happens, AI should be considered as a tool that can only assist a physician in the diagnostic and prognostic pathway of ACS, without ever replacing the human being in the final decision, which must be made based on the personal medical experience of the physician and on critical thinking, features that do not yet fully characterize AI systems.

## References

[1] Townsend N., Kazakiewicz D., Lucy Wright F., Timmis A., Huculeci R., Torbica A., Gale C.P., Achenbach S., Weidinger F., Vardas P. (2021). Epidemiology of cardiovascular disease in Europe. Nat. Rev. Cardiol..

[2] Timmis A., Vardas P., Townsend N., Torbica A., Katus H., De Smedt D., Gale C.P., Maggioni A.P., Petersen S.E., Huculeci R. (2022). European Society of Cardiology: Cardiovascular disease statistics 2021. Eur. Heart J..

[3] Timmis A., Kazakiewicz D., Townsend N., Huculeci R., Aboyans V., Vardas P. (2023). Global epidemiology of acute coronary syndromes. Nat. Rev. Cardiol..

[4] Benjamin E.J., Blaha M.J., Chiuve S.E., Cushman M., Das S.R., Deo R., de Ferranti S.D., Floyd J., Fornage M., Gillespie C. (2017). Heart Disease and Stroke Statistics-2017 Update: A Report From the American Heart Association. Circulation.

[5] Beiser D.G., Cifu A.S., Paul J. (2022). Evaluation and Diagnosis of Chest Pain 2022. JAMA.

[6] Agzew Y. (2009). Elevated serum cardiac troponin in non-acute coronary syndrome. Clin. Cardiol..

[7] Kilic A. (2020). Artificial Intelligence and Machine Learning in Cardiovascular Health Care. Ann. Thorac. Surg..

[8] Baethge C., Goldbeck-Wood S., Mertens S. (2019). SANRA—A scale for the quality assessment of narrative review articles. Res. Integr. Peer. Rev..

[9] Byrne R.A., Rossello X., Coughlan J.J., Barbato E., Berry C., Chieffo A., Claeys M.J., Dan G.A., Dweck M.R., Galbraith M. (2023). 2023 ESC Guidelines for the management of acute coronary syndromes Developed by the task force on the management of acute coronary syndromes of the European Society of Cardiology (ESC) Ibanez * †, (Chairperson) (Spain), and ESC Scientific Document Group 2023. Eur. Heart J..

[10] Thygesen K., Alpert J.S., Jaffe A.S., Chaitman B.R., Bax J.J., Morrow D.A., White H.D. (2018). Fourth Universal Definition of Myocardial Infarction (2018). Circulation.

[11] Bhatt D.L., Lopes R.D., Harrington R.A. (2022). Diagnosis and Treatment of Acute Coronary Syndromes: A Review. JAMA.

[12] Diercks D.B., Peacock W.F., Hiestand B.C., Chen A.Y., Pollack C.V., Kirk J.D., Smith S.C., Gibler W.B., Ohman E.M., Blomkalns A.L. (2006). Frequency and consequences of recording an electrocardiogram >10 minutes after arrival in an emergency room in non-ST-segment elevation acute coronary syndromes (from the CRUSADE initiative). Am. J. Cardiol..

[13] De Luca G., Suryapranata H., Ottervanger J.P., Antman E.M. (2004). Time Delay to Treatment and Mortality in Primary Angioplasty for Acute Myocardial Infarction. Circulation.

[14] Shah A.S.V., Anand A., Strachan F.E., Ferry A.V., Lee K.K., Chapman A.R., Sandeman D., Stables C.L., Adamson P.D., Andrews J.P.M. (2018). High-sensitivity troponin in the evaluation of patients with suspected acute coronary syndrome: A stepped-wedge, cluster-randomised controlled trial. Lancet.

[15] Neumann J.T., Sörensen N.A., Schwemer T., Ojeda F., Bourry R., Sciacca V., Schaefer S., Waldeyer C., Sinning C., Renné T. (2016). Diagnosis of Myocardial Infarction Using a High-Sensitivity Troponin I 1-Hour Algorithm. JAMA Cardiol..

[16] Birnbaum Y., Bayés de Luna A., Fiol M., Nikus K., Macfarlane P., Gorgels A., Sionis A., Cinca J., Barrabes J.A., Pahlm O. (2012). Common pitfalls in the interpretation of electrocardiograms from patients with acute coronary syndromes with narrow QRS: A consensus report. J. Electrocardiol..

[17] Cook D.A., Oh S.Y., Pusic M.V. (2020). Accuracy of Physicians’ Electrocardiogram Interpretations: A Systematic Review and Meta-analysis. JAMA Intern. Med..

[18] Lai Y.C., Chen Y.H., Wu K.H., Chen Y.C. (2020). Validation of the diagnosis and triage algorithm for acute myocardial infarction in the setting of left bundle branch block. Am. J. Emerg. Med..

[19] Nestelberger T., Cullen L., Lindahl B., Reichlin T., Greenslade J.H., Giannitsis E., Christ M., Morawiec B., Miro O., Martín-Sánchez F.J. (2019). Diagnosis of acute myocardial infarction in the presence of left bundle branch block. Heart.

[20] Wang T.Y., Zhang M., Fu Y., Armstrong P.W., Newby L.K., Gibson C.M., Moliterno D.J., Van de Werf F., White H.D., Harrington R.A. (2009). Incidence, distribution, and prognostic impact of occluded culprit arteries among patients with non-ST-elevation acute coronary syndromes undergoing diagnostic angiography. Am. Heart J..

[21] Khan A.R., Golwala H., Tripathi A., Bin Abdulhak A.A., Bavishi C., Riaz H., Mallipedi V., Pandey A., Bhatt D.L. (2017). Impact of total occlusion of culprit artery in acute non-ST elevation myocardial infarction: A systematic review and meta-analysis. Eur. Heart J..

[22] Niu T., Fu P., Jia C., Dong Y., Liang C., Cao Q., Yang Z., Fu R., Zhang X., Sun Z. (2013). The delayed activation wave in non-ST-elevation myocardial infarction. Int. J. Cardiol..

[23] De Winter R.J., Verouden N.J.W., Wellens H.J.J., Wilde A.A.M. (2008). A New ECG Sign of Proximal LAD Occlusion. N. Engl. J. Med..

[24] Boeddinghaus J., Nestelberger T., Twerenbold R., Neumann J.T., Lindahl B., Giannitsis E., Sörensen N.A., Badertscher P., Jann J.E., Wussler D. (2018). Impact of age on the performance of the ESC 0/1h-algorithms for early diagnosis of myocardial infarction. Eur. Heart J..

[25] Hillinger P., Twerenbold R., Wildi K., Rubini Gimenez M., Jaeger C., Boeddinghaus J., Nestelberger T., Grimm K., Reichlin T., Stallone F. (2017). Gender-specific uncertainties in the diagnosis of acute coronary syndrome. Clin. Res. Cardiol..

[26] Twerenbold R., Badertscher P., Boeddinghaus J., Nestelberger T., Wildi K., Puelacher C., Sabti Z., Rubini Gimenez M., Tschirky S., du Fay de Lavallaz J. (2018). 0/1-Hour Triage Algorithm for Myocardial Infarction in Patients with Renal Dysfunction. Circulation.

[27] Anand A., Lee K.K., Chapman A.R., Ferry A.V., Adamson P.D., Strachan F.E., Berry C., Findlay I., Cruikshank A., Reid A. (2021). High-Sensitivity Cardiac Troponin on Presentation to Rule Out Myocardial Infarction: A Stepped-Wedge Cluster Randomized Controlled Trial. Circulation.

[28] Reichlin T., Twerenbold R., Reiter M., Steuer S., Bassetti S., Balmelli C., Winkler K., Kurz S., Stelzig C., Freese M. (2012). Introduction of high-sensitivity troponin assays: Impact on myocardial infarction incidence and prognosis. Am. J. Med..

[29] Karatzia L., Aung N., Aksentijevic D. (2022). Artificial intelligence in cardiology: Hope for the future and power for the present. Front. Cardiovasc. Med..

[30] Makimoto H., Kohro T. (2023). Adopting artificial intelligence in cardiovascular medicine: A scoping review. Hypertens. Res..

[31] Al-Zaiti S., Besomi L., Bouzid Z., Faramand Z., Frisch S., Martin-Gill C., Gregg R., Saba S., Callaway C., Sejdić E. (2020). Machine learning-based prediction of acute coronary syndrome using only the pre-hospital 12-lead electrocardiogram. Nat. Commun..

[32] Chen K.W., Wang Y.C., Liu M.H., Tsai B.Y., Wu M.Y., Hsieh P.H., Wei J.T., Shih E.S.C., Shiao Y.T., Hwang M.J. (2022). Artificial intelligence-assisted remote detection of ST-elevation myocardial infarction using a mini-12-lead electrocardiogram device in prehospital ambulance care. Front. Cardiovasc. Med..

[33] Zhao Y., Xiong J., Hou Y., Zhu M., Lu Y., Xu Y., Teliewubai J., Liu W., Xu X., Li X. (2020). Early detection of ST-segment elevated myocardial infarction by artificial intelligence with 12-lead electrocardiogram. Int. J. Cardiol..

[34] Choi Y.J., Park M.J., Ko Y., Soh M.S., Kim H.M., Kim C.H., Lee E., Kim J. (2022). Artificial intelligence versus physicians on interpretation of printed ECG images: Diagnostic performance of ST-elevation myocardial infarction on electrocardiography. Int. J. Cardiol..

[35] Herman R., Meyers H.P., Smith S.W., Bertolone D.T., Leone A., Bermpeis K., Viscusi M.M., Belmonte M., Demolder A., Boza V. (2024). International evaluation of an artificial intelligence–powered electrocardiogram model detecting acute coronary occlusion myocardial infarction. Eur. Heart J. Digit. Health.

[36] Liu W.C., Lin C.S., Tsai C.S., Tsao T.P., Cheng C.C., Liou J.T., Lin W.S., Cheng S.M., Lou Y.S., Lee C.C. (2021). A deep learning algorithm for detecting acute myocardial infarction. EuroIntervention.

[37] Wu C.C., Hsu W.D., Islam M.M., Poly T.N., Yang H.C., Nguyen P.A., Wang Y.C., Li Y.J. (2019). An artificial intelligence approach to early predict non-ST-elevation myocardial infarction patients with chest pain. Comput. Methods Programs Biomed..

[38] Qin L., Qi Q., Aikeliyaer A., Hou W.Q., Zuo C.X., Ma X. (2022). Machine learning algorithm can provide assistance for the diagnosis of non-ST-segment elevation myocardial infarction. Postgrad. Med. J..

[39] Berikol G.B., Yildiz O., Özcan T. (2016). Diagnosis of Acute Coronary Syndrome with a Support Vector Machine. J. Med. Syst..

[40] Than M.P., Pickering J.W., Sandoval Y., Shah A.S.V., Tsanas A., Apple F.S., Blankenberg S., Cullen L., Mueller C., Neumann J.T. (2019). Machine Learning to Predict the Likelihood of Acute Myocardial Infarction. Circulation.

[41] Doudesis D., Lee K.K., Boeddinghaus J., Bularga A., Ferry A.V., Tuck C., Lowry M.T.H., Lopez-Ayala P., Nestelberger T., Koechlin L. (2023). Machine learning for diagnosis of myocardial infarction using cardiac troponin concentrations. Nat. Med..

[42] Kayvanpour E., Gi W.T., Sedaghat-Hamedani F., Lehmann D.H., Frese K.S., Haas J., Tappu R., Samani O.S., Nietsch R., Kahraman M. (2021). microRNA neural networks improve diagnosis of acute coronary syndrome (ACS). J. Mol. Cell. Cardiol..

[43] Ulvenstam A., Graipe A., Irewall A.L., Söderström L., Mooe T. (2023). Incidence and predictors of cardiovascular outcomes after acute coronary syndrome in a population-based cohort study. Sci. Rep..

[44] Khera R., Haimovich J., Hurley N.C., McNamara R., Spertus J.A., Desai N., Rumsfeld J.S., Masoudi F.A., Huang C., Normand S.L. (2021). Use of Machine Learning Models to Predict Death After Acute Myocardial Infarction. JAMA Cardiol..

[45] Hadanny A., Shouval R., Wu J., Shlomo N., Unger R., Zahger D., Matetzky S., Goldenberg I., Beigel R., Gale C. (2021). Predicting 30-day mortality after ST elevation myocardial infarction: Machine learning- based random forest and its external validation using two independent nationwide datasets. J. Cardiol..

[46] Sherazi S.W.A., Jeong Y.J., Jae M.H., Bae J.W., Lee J.Y. (2020). A machine learning-based 1-year mortality prediction model after hospital discharge for clinical patients with acute coronary syndrome. Health Inform. J..

[47] Sherazi S.W.A., Bae J.W., Lee J.Y. (2021). A soft voting ensemble classifier for early prediction and diagnosis of occurrences of major adverse cardiovascular events for STEMI and NSTEMI during 2-year follow-up in patients with acute coronary syndrome. PLoS ONE.

[48] D’Ascenzo F., De Filippo O., Gallone G., Mittone G., Deriu M.A., Iannaccone M., Ariza-Solé A., Liebetrau C., Manzano-Fernández S., Quadri G. (2021). Machine learning-based prediction of adverse events following an acute coronary syndrome (PRAISE): A modelling study of pooled datasets. Lancet.

[49] Mohammad M.A., Olesen K.K.W., Koul S., Gale C.P., Rylance R., Jernberg T., Baron T., Spaak J., James S., Lindahl B. (2022). Development and validation of an artificial neural network algorithm to predict mortality and admission to hospital for heart failure after myocardial infarction: A nationwide population-based study. Lancet Digit. Health.

[50] Lee W., Lee J., Woo S.I., Choi S.H., Bae J.W., Jung S., Jeong M.H., Lee W.K. (2021). Machine learning enhances the performance of short and long-term mortality prediction model in non-ST-segment elevation myocardial infarction. Sci. Rep..

[51] Shavadia J.S., Chen A.Y., Fanaroff A.C., de Lemos J.A., Kontos M.C., Wang T.Y. (2019). Intensive Care Utilization in Stable Patients With ST-Segment Elevation Myocardial Infarction Treated With Rapid Reperfusion. JACC Cardiovasc. Interv..

